# Retrieval of a migrated lumen-apposing metal stent from a pancreatic fluid collection using a balloon enteroscopy overtube

**DOI:** 10.1055/a-2901-6548

**Published:** 2026-07-08

**Authors:** Yuki Tanisaka, Shomei Ryozawa, Masafumi Mizuide, Akashi Fujita, Suguru Ito, Ryuichi Watanabe, Yoshiki Matsuno

**Affiliations:** 1 Department of Gastroenterology 183786 Saitama Medical University International Medical CenterHidakaSaitama PrefectureJapan

## A case description



**Video 1** Successful retrieval of a migrated lumen-apposing metal stent (LAMS) from a pancreatic fluid collection (PFC) using a balloon enteroscopy overtube.


Endoscopic ultrasound (EUS)-guided drainage of a pancreatic fluid collection (PFC) using a lumen-apposing metal stent (LAMS) has been reported to achieve high technical and clinical success rates and is now widely used. 1​ 2​ ​3​ However, stent migration can occur, and surgical intervention may be required if endoscopic retrieval is unsuccessful. 4​ We report a case of successful retrieval of a migrated LAMS from the PFC using a balloon enteroscopy overtube.

**Fig. 1:**
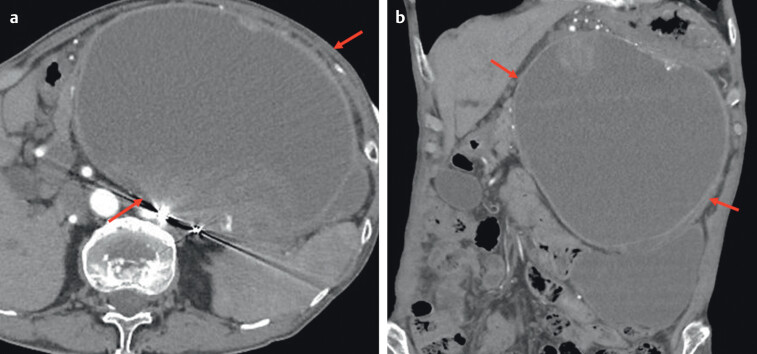
Computed tomography revealing pancreatic fluid collection measuring more than 10 cm in diameter (red arrow).

**Fig. 2:**
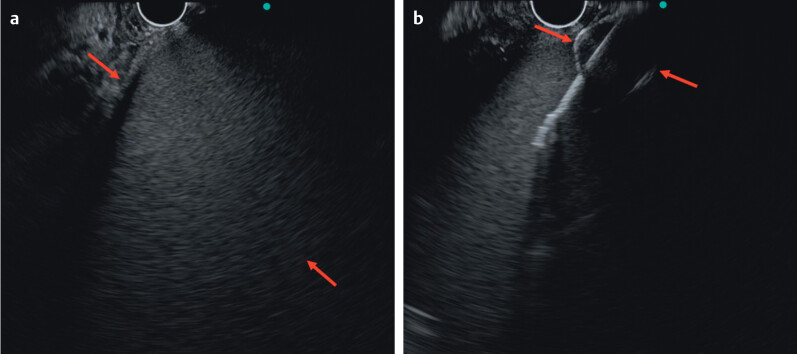
Endoscopic ultrasound (EUS) findings. ( **a** ). EUS revealing the pancreatic fluid collection (PFC) measuring more than 10 cm in diameter (red arrow). ( **b** ) A 15-mm lumen-apposing metal stent (red arrow) was deployed for drainage of the PFC.

**Fig. 3:**
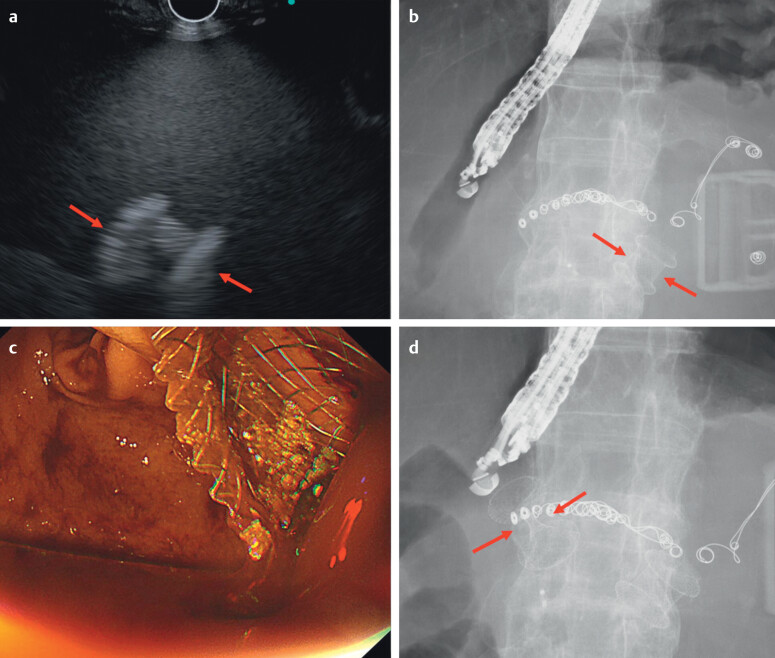
Endoscopic ultrasound (EUS) and fluoroscopic findings. ( **a** and **b** ) Migration of the lumen-apposing metal stent (LAMS) into the pancreatic fluid collection was detected upon EUS and fluoroscopic imaging (red arrow). ( **c** and **d** ) Another LAMS (red arrow) was immediately deployed to maintain drainage and prevent worsening peritonitis.

**Fig. 4:**
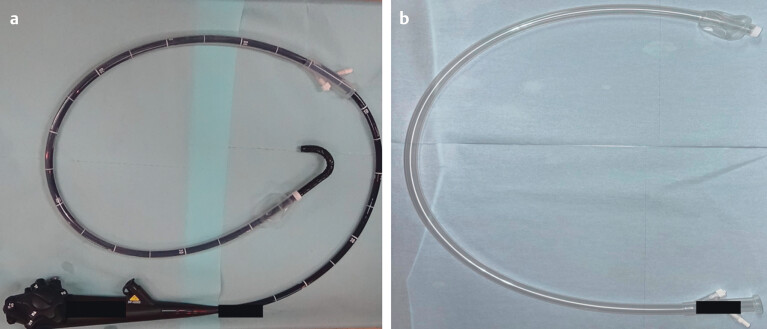
Short-type single-balloon enteroscope (short SBE; SIF-H290; Olympus Marketing, Japan) with a working length of 152 cm and a 3.2-mm working channel and its overtube with 11-mm inner diameter.

**Fig. 5:**
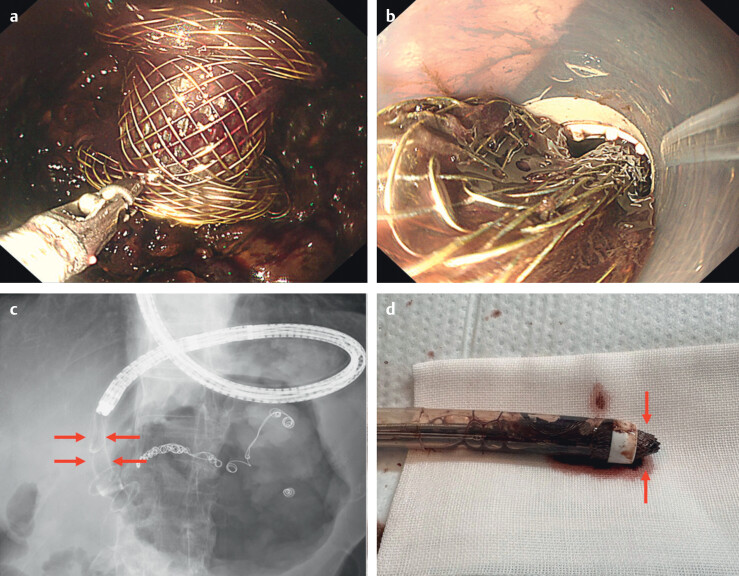
Endoscopic and fluoroscopic findings. ( **a** ) The migrated lumen-apposing metal stent (LAMS) was grasped using rat-tooth forceps. ( **b** and **c** ) The migrated LAMS was pulled into the overtube (red arrow) by withdrawing only the short SBE. ( **d** ). The migrated LAMS (red arrow) was safely retrieved without damaging the deployed LAMS, esophagogastric junction, or pharynx.

An 84-year-old man with PFC secondary to alcohol-induced pancreatitis was referred to our institution for treatment ( Fig. 1 ). EUS-guided drainage of the PFC using a LAMS was planned ( Video 1 ). A PFC measuring more than 10 cm in diameter was visualized from the stomach using a linear echoendoscope (UCT-260; Olympus Marketing, Japan), and a 15-mm LAMS was deployed for drainage of the PFC ( Fig. 2 ). However, migration of the LAMS into the PFC was detected upon EUS and fluoroscopic imaging. Another 15-mm LAMS was immediately deployed to maintain drainage and prevent worsening peritonitis ( Fig.3 ). Two weeks later, endoscopic retrieval of the migrated LAMS was attempted using a short-type single-balloon enteroscope (short SBE; SIF-H290; Olympus Marketing, Japan) with a working length of 152 cm and a 3.2-mm working channel and its overtube with 11-mm inner diameter ( Fig.4 ). 5​ The short SBE was advanced into the PFC through the deployed LAMS and the migrated LAMS was identified. The migrated LAMS was grasped using rat-tooth forceps and pulled into the overtube by withdrawing only the short SBE. Finally, the migrated LAMS was safely retrieved by simultaneously withdrawing the short SBE and overtube without damaging the deployed LAMS, esophagogastric junction, or pharynx ( Fig. 5 ).

This case highlights the feasibility and safety of retrieving a migrated LAMS using a balloon enteroscopy overtube, which may represent an effective option for managing the migrated LAMS in such cases.

Endoscopy_UCTN_Code_TTT_1AS_2AK
